# Renal Toxicity of Rosuvastatin: A Case Report

**DOI:** 10.7759/cureus.96013

**Published:** 2025-11-03

**Authors:** Carl Schulze, Michael Shye

**Affiliations:** 1 Nephrology, University of California Los Angeles, Los Angeles, USA; 2 Medicine/Nephrology, University of California Los Angeles, Los Angeles, USA

**Keywords:** acute kidney injury, acute tubular necrosis (atn), high-statin therapy, prevention of cardiovascular disease, statin safety

## Abstract

This is a report of a 64-year-old man who developed acute kidney injury (AKI) associated with high-dose rosuvastatin. He had been treated with statins for several years due to coronary artery disease, hypertension, and hyperlipidemia. He had initially been treated with simvastatin, though it was changed to rosuvastatin for stronger effects on cholesterol and cardiovascular risk reduction. About 12 months after getting rosuvastatin 40 mg per day, AKI was detected. Though other factors were likely contributing, the AKI appeared to be nephrotoxic in nature. There were two medications on his list, rosuvastatin and pantoprazole, which plausibly could cause reduced kidney function, and we discussed a trial of medication withdrawal. He had severe esophageal disease, which was at risk of a flare with coming off the pantoprazole, so we elected to stop rosuvastatin first. After stopping rosuvastatin, the kidney function returned to baseline, and the abnormal urine testing resolved.

## Introduction

Using statin medications to prevent cardiovascular (CV) events represents a major medical advancement since lovastatin was first approved in the United States in 1987 [1]. Patients with chronic kidney disease (CKD) have a high risk of CV disease, and the 2024 Kidney Disease: Improving Global Outcomes (KDIGO) CKD guidelines recommend starting statins in most patients not on dialysis [2]. In addition to preventing CV events in patients with kidney diseases, statins are the standard of care to treat hypercholesterolemia in nephrotic syndrome. They are well tolerated in most patients, with only a small minority experiencing side effects of myopathy (1-10 percent (%)) and transaminitis (1%). The renal risks are even lower and mostly limited to case reports of acute kidney injury (AKI) associated with rhabdomyolysis (<0.1%) [3]. Hence, statins are a common entry on medication lists of patients referred to nephrology and are rarely a concern when patients suffer unexplained declines in kidney function.

However, rosuvastatin has been associated with higher rates of renal toxicity than other statins in several reports [4]. Preapproval data for rosuvastatin showed proteinuria and hematuria in a dose-dependent manner, with rates less than 1% at the daily doses of 10 and 20 mg, 1.3% at 40 mg/d, and about 6% of patients at the 80 mg/d dose, part of the reason this dose was not pursued further [5]. The mechanism of toxicity was unclear, but “the suggestion of tubular inflammation and necrosis” was reported [5]. The urinary abnormalities were associated with more severe renal effects, as 41% of patients with proteinuria or hematuria at the 80 mg/d dose had an increase in creatinine (Cr) of >30% [5]. The possibility of AKI resulting from statin use is a concern, given that AKI is associated with increased morbidity, mortality, cost, and risk of CKD [6].

## Case presentation

The patient is a 64-year-old male referred to nephrology due to elevated Cr. His past medical history included hypertension, hyperlipidemia, aortic regurgitation, coronary artery disease without angina, eosinophilic esophagitis, and migraine headaches. The current medications were vitamin C 1,000 mg/d, carvedilol 3.125 mg twice daily, diazepam 5 mg as needed, budesonide suspension 2 mg twice daily, fish oil 1 g daily, fluticasone inhaler 220 mcg twice a day, fluticasone nasal spray one spray each nostril daily, gabapentin 100 mg three times daily, multivitamin one tab daily, olopatadine ophthalmic solution 0.1% twice a day, pantoprazole 20 mg/d, rimegepant 75 mg every other day, rosuvastatin 40 mg/d, and zinc sulfate 50 mg/d (rosuvastatin is not known to interact with these other medications). He had previously been prescribed simvastatin for primary prevention of CV disease by his cardiologist, whom he was seeing for mild aortic regurgitation and atypical chest pain. Roughly 16 months before developing AKI, his simvastatin was changed to rosuvastatin 20 mg/d for stronger lowering of low-density lipoprotein (LDL). The dose was then increased to 40 mg/d six months later due to mild coronary artery disease seen on computed tomography (CT) angiogram of the coronary arteries, thoracic aorta, and abdominal aorta.

As shown in Table 1, the baseline Cr was 0.9-1.0 mg/dL, and one month after changing to rosuvastatin 20 mg/d, Cr was 1.4 mg/dL, though three months later had returned to 1.0 mg/dL. Two months into taking rosuvastatin 40 mg/d, the Cr was still at baseline. Then, there was a gap in labs for 12 months until he was admitted with syncope and influenza A to a hospital (by this time, he had been on rosuvastatin 40 mg/d for 14 months). He had reported a week of flu-like illness, poor oral intake, nausea, vomiting, and use of ibuprofen along with his prescription medications. On admission, Cr was elevated to 2.2 mg/dL. Urinalysis showed 1+ protein and 1+ blood on dipstick, microscopy was negative, random urine sodium was 118 mmol/L, and ultrasound showed reduced sizes of the kidneys (about 8 cm each side), no hydronephrosis, and a simple cyst on the right kidney. Over the three-day hospital stay, he was treated supportively, including with IV fluids, and the Cr fell to 1.4 mg/dL at discharge. One week later, at the post-hospitalization appointment, it was 1.3 mg/dL, but then, four weeks later, it had increased to 1.6 mg/dL when he was referred to nephrology for a consultation.

**Table 1:** Summary of creatinine, EGFR, blood pressure, and changes to medications Cr: creatinine; EGFR: estimated glomerular filtration rate; BP: blood pressure; HCTZ: hydrochlorothiazide; mg/dL: milligrams per deciliter; mL/min: milliliters per minute; mmHg: millimeters of mercury

Date	Cr (mg/dL)	EGFR (mL/min/1.73 m^2^)	BP (mmHg)	Comment
10/28/2022	0.92	>89	123/78	Baseline
5/30/2023	NA	NA	135/84	Started rosuvastatin 20 mg/d
6/21/2023	1.41	56	NA	Had taken HCTZ for vertigo
10/4/2023	1.00	85	NA	
10/16/2023	NA	NA	132/83	Rosuvastatin increased to 40 mg/d
12/29/2023	1.02	83	132/83	
12/26/2024	2.17	33	116/66	Admitted for influenza A
12/29/2024	1.44	55	120/75	Discharge
1/6/2025	1.29	62	127/78	One week post-discharge
1/13/2025	1.35	59	134/72	
1/31/2025	1.63	47	143/77	Referred to nephrology
2/5/2025	1.58	49	129/79	
3/17/2025	1.31	61	129/75	
4/8/2025	1.71	44	134/78	
5/5/2025	1.58	49	NA	Crestor stopped 2 d later
5/14/2025	NA	NA	114/74	
5/23/2025	1.24	65	NA	
5/27/2025	NA	NA	118/65	
6/4/2025	1.13	73	144/82	
7/3/2025	1.20	68	127/76	
9/9/2025	1.20	68	119/75	

At the initial appointment, he had no acute complaints and a normal exam. Review of systems was negative, including no swelling, shortness of breath, skin rash, difficulty passing urine, bloody urine, dark urine, or myalgias. Vital signs were temp 36.7°C, pulse 70, blood pressure 129/79, weight 61.7 kg, and BMI 23.4. A repeat kidney ultrasound showed normal sizes of the kidneys and again a simple appearing cyst on the right. As indicated in Table 2, urinalysis showed 1+ protein, trace blood, granular casts, urine albumin to Cr ratio of 184 mg/g, and total protein to Cr ratio of 0.7 g/g. Over the next three months, the kidney function remained stable. Red blood cells (RBC) appeared on urine microscopy in two of the four urine samples, and low-grade proteinuria persisted. No dysmorphic RBC or RBC casts were seen. The only other lab abnormality was mildly elevated alkaline phosphatase (AP), along with bone-specific AP (less than two times the upper limit of normal). Serum creatine kinase (CK) was not checked. A summary of the changes in urinalysis corresponding to rosuvastatin and admission for influenza is shown in Table 2.

**Table 2:** Urine testing on select dates ACR: albumin/creatinine ratio; UPC: urine protein/creatinine ratio; mg/g: milligrams albumin per gram of creatinine; g/g: grams protein per gram of creatinine; RBC: red blood cells; HPF: high-powered field

Date	Urine protein	Urine microscopy	Comments
10/28/2022	Negative	Normal limits	
12/29/2023	Trace	Normal limits	Rosuvastatin at 40 mg/g
12/27/2024	1+	1+ blood, 0-5 RBC/HPF	Admitted for influenza A
2/5/2025	1+; ACR 184 mg/g, UPC 0.7 g/g	Trace blood, 1 RBC/HPF, granular casts	
3/17/2025	1+; UPC 0.9 g/g	1 RBC/HPF	
4/8/2025	1+ protein; ACR 280 mg/g	15 RBC/HPF	
5/5/2025	2+ protein; UPC 0.8 g/g	16 RBC/HPF	Rosuvastatin stopped 2 d later
6/23/2025	Negative dipstick, UPC negative	0 RBC/HPF	

He underwent additional noninvasive evaluation for CKD. Urine protein electrophoresis showed monoclonal protein but negative immunofixation, and serum protein electrophoresis and immunofixation were negative. A myeloma bone survey was negative, and repeat urine protein electrophoresis was negative. He subsequently underwent hematology evaluation and was deemed most likely to have had a false-positive urine monoclonal protein test. Hemoglobin a1c was 5.4 on October 4, 2023, and fasting blood glucose was less than 100 on multiple subsequent labs. Total cholesterol was 157 mg/dL, high-density lipoprotein (HDL) 59 mg/dL, LDL 74 mg/dL, and triglycerides (TG) 121 mg/dL. Serologies showed negative antineutrophil cytoplasmic antibody (ANCA), antinuclear antibody (ANA), and anti-double-stranded DNA (dsDNA), and complement levels C3 and C4 were within the normal range. Infectious serologies showed negative rapid plasma reagent (RPR), hepatitis C antibody, and human immunodeficiency virus (HIV) antibody. Hepatitis B (HB) serology showed negative HB surface antigen and HB core antibody and positive HB surface antibody, consistent with prior vaccination.

Over the next three months, the Cr remained elevated between 1.5 and 1.7 mg/dL, and the cystatin C was 1.3-1.4 mg/dL until May 5, 2025. The patient and I then discussed obtaining a kidney biopsy and, in the meantime, stopping medications, which could be causing AKI. We focused on rosuvastatin and pantoprazole; as he had been on proton pump inhibitor (PPI) therapy for more than five years, the pantoprazole was less likely to be causing this more recent AKI, and there was a risk of causing an exacerbation of the esophagitis. Hence, we opted to try to stop the rosuvastatin first. Three weeks later, the Cr fell to 1.2 mg/dL and has remained there on subsequent lab testing (Table 1). The urine abnormalities resolved, with no proteinuria or hematuria and a negative microscopy. After improvement in kidney function, we rediscussed the risk-benefit analysis of proceeding with a kidney biopsy, and he preferred to avoid the risk of bleeding, so the biopsy was cancelled.

## Discussion

Over the past few decades, the pleiotropic effects of statins, or those other than the cholesterol-lowering effects, have been studied in how they affect renal outcomes. Pravastatin, lovastatin, and simvastatin were shown to reduce urine protein excretion in patients with hypertension, diabetes, and immunoglobulin A (IGA) nephropathy, but fluvastatin was associated with new-onset hematuria and proteinuria in a hyperlipidemic patient [4]. Statin therapy was associated in an observational study with reduced risk of AKI in patients admitted to a single hospital in Italy with COVID-19 pneumonia [7]. Though animal studies suggested some renal benefits to statins via improved endothelial cell function and reduced oxidative stress, trials using them to prevent AKI in cardiac surgery patients failed to show the benefit of treatment [8]. The recent Statin Use in Cardiac Surgery (STICS) trial attempted to use rosuvastatin to lower the risk of postoperative atrial fibrillation in patients undergoing cardiac surgery, but found a higher risk of AKI (in addition to no prevention of atrial fibrillation rate) in the treatment arm [9].

Nephrotoxicity has been seen in additional settings. Most concern has focused on rhabdomyolysis-associated AKI [3], but rosuvastatin has been associated with AKI without rhabdomyolysis in several reports, including the above trial in the perioperative setting [9]. A case report from 2004 described a patient in South Africa who developed AKI, proteinuria, and abnormal urine sediment not associated with rhabdomyolysis 18 months after changing atorvastatin 80 mg/d to rosuvastatin 80 mg/d, a dose which never received FDA approval [10]. A kidney biopsy showed acute and chronic interstitial nephritis and increased size of proximal tubular cell mitochondria on electron microscopy [10]. The AKI and urine abnormalities resolved after cessation of rosuvastatin, though he was rechallenged at the same dose, and the urine abnormalities recurred. Treatment was then changed to atorvastatin 40 mg/d, and urine abnormalities improved, though did not resolve until changing to simvastatin 20 mg/d [10]. Another report of tubular toxicity ascribed to rosuvastatin, published in 2017, described a patient treated with rosuvastatin 40 mg/d who experienced proteinuria, microhematuria, and increased Cr from a baseline of 0.7 to 1.8 [11]. Rosuvastatin was stopped, the Cr quickly trended down, and she underwent a kidney biopsy showing acute tubular injury (the histological correlate to acute tubular necrosis (ATN)). Her Cr eventually improved to 0.9, and proteinuria and hematuria resolved [11]. While several national registry monitoring programs did not show increased risk of AKI in patients prescribed rosuvastatin [12], a large retrospective study of healthcare organizations in 2022 found a mildly increased risk of proteinuria, hematuria, and AKI in patients newly prescribed rosuvastatin compared to atorvastatin and noted the risks were dose-dependent [13]. Luckily, all reported cases of AKI described above showed recovery of kidney function and urine abnormalities upon cessation of the medication [4,9-12].

**Figure 1:**
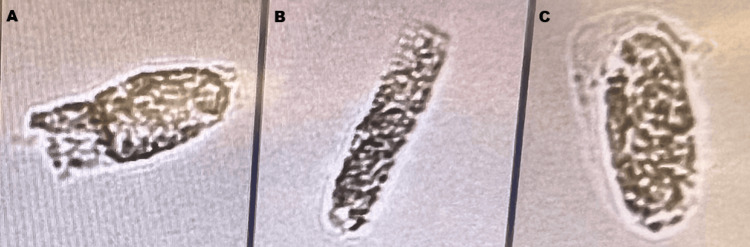
Granular casts (A-C) Granular casts as seen in a urine sediment, using the IQ200 automated urinalysis instrument with Automated Particle Recognition software (Beckman Coulter, Brea, CA, US). Magnification is approximately 20 times. Used with permission from the teaching file of Lu Song, Ph.D.

The reports all point to the proximal tubule as the site of injury in statin-associated AKI (not due to rhabdomyolysis). Evidence for proximal tubular toxicity is supported by the increased size of the proximal tubular mitochondria on the biopsy by van Zyl-Smit et al. [10] and acute tubular injury by Ward et al. [11], though the earlier biopsy showed evidence of immune-mediated injury as well. A biomarker of renal tubular injury, Kidney Injury Molecule-1 (KIM-1) [14], was significantly more elevated in the rosuvastatin arm compared to the placebo arm of the STICS trial [9]. In a similar vein, the patient in the case had markers of proximal tubular toxicity: urine granular or muddy brown casts, as in Figure 1 below, and the pattern of a more marked elevation in urine total protein/creatine compared to a more modest elevation of urine albumin/Cr [15,16].

In summary, the patient above suffered AKI related to rosuvastatin, initially detected when he was admitted to a hospital for influenza, though it persisted after adequate treatment of that condition, which included IV fluids and withholding of all nonsteroidal anti-inflammatory drugs (NSAIDs). He had evidence for ATN based on the reduced kidney function, granular casts on urinalysis, and an elevated ratio of urine total protein to albumin. Given that the kidney injury began after taking the medication, there are previous reports of this adverse effect, improvement occurred after discontinuation, the reaction was not seen at a lower dose, and objective evidence of toxicity was present (reduced kidney function and urinary abnormalities), this would qualify as a probable adverse drug reaction on the Naranjo scale [17]. Nonetheless, other causes of ATN were present, namely, influenza, NSAID use, and volume depletion, and he is also taking chronic PPI, which are a known cause of late-onset AKI or CKD. Hence, these other causes remain relevant, and ongoing follow-up is needed.

## Conclusions

This patient suffered AKI with rosuvastatin as a probable cause. ATN is often multifactorial in cause, so it is possible that influenza, volume depletion, NSAID use, and rosuvastatin all combined to trigger ATN, which then persisted after the other prior insults had been addressed. His clinical picture fits with prior reports of rosuvastatin-associated AKI, with intermittent microhematuria and evidence for tubular toxicity with a relatively higher urine total protein/Cr compared with a more modest elevation of urine albumin/Cr ratios. Fortunately, like in the prior reports, his ATN resolved with the cessation of the medication. As the AKI due to rosuvastatin remains rare, it is unclear if serial monitoring of kidney function is needed for all patients taking the medication, though increased awareness of the potential risk of AKI could help lead to earlier detection and more prompt changes to therapy. Future research into the mechanism of tubular injury due to rosuvastatin would be helpful.
